# Maternal anthropometric characteristics in pregnancy and blood pressure among adolescents: 1993 live birth cohort, Pelotas, southern Brazil

**DOI:** 10.1186/1471-2458-10-434

**Published:** 2010-07-23

**Authors:** Helen C Laura, Ana B Menezes, Ricardo B Noal, Pedro C Hallal, Cora L Araújo

**Affiliations:** 1 Post-Graduate Program in Epidemiology, Federal University of Pelotas, CP 464, 96001-970, Pelotas, RS, Brazil

## Abstract

**Background:**

We investigated the association between maternal anthropometric measurements in prepregnancy and at the end of pregnancy and their children's systolic (SBP) and diastolic (DBP) blood pressure at 11 years of age, in a prospective cohort study.

**Methods:**

All hospital births which took place in 1993 in the city of Pelotas - Brazil, were identified (5,249 live births). In 2004, the overall proportion of follow-up was 85% and we obtained arterial blood pressure measurements of 4,452 adolescents.

**Results:**

Independent variables analyzed included maternal prepregnancy weight and body mass index (BMI) and maternal weight, and height at the end of pregnancy. Multiple linear regression analysis controlling for the following confounders were carried out: adolescent's skin color, family income at birth, smoking, alcohol intake during pregnancy, and gestational arterial hypertension. Mean SBP and DBP were 101.9 mmHg (SD 12.3) and 63.4 mmHg (SD 9.9), respectively. Maternal prepregnancy weight and BMI, and weight at the end of pregnancy were positively associated with both SBP and DBP in adolescent subjects of both sexes; maternal height was positively associated with SBP only among males.

**Conclusions:**

Adequate evaluation of maternal anthropometric characteristics during pregnancy may prevent high levels of blood pressure among adolescent children.

## Background

Barker's hypothesis of the fetal origin of chronic diseases suggests that early-life determinants - such as adverse conditions in intrauterine life or during early childhood - can trigger adaptations in the fetus or child which may predispose to the development of systemic arterial hypertension and coronary cardiovascular disease in adult life [1,2].

In 1994, Godfrey et al [3] found poor maternal nutrition during pregnancy to be associated with high blood pressure among 10-12-year-old children. Laor et al [4], in a study of maternal anthropometric variables and blood pressure among their adolescent children, found that increased maternal prepregnancy weight and body mass index (BMI) were both associated with increased systolic and diastolic blood pressure. Regarding mother's height, Adair et al [5] found an inverse association between this variable and systolic blood pressure (SBP) among adolescents.

There is evidence that the precursors of atherosclerosis and cardiovascular disease originate during childhood [6]. High blood pressure during childhood and adolescents are the major predictive factor for development of hypertension in adult life, and are associated with different forms of cardiovascular disease [7]. A small increase in blood pressure at an early age can have important effects later in life; Munter et al [8], for instance, report that every 1-2 mm Hg increase in SBP during childhood corresponds to a 10% increase in risk of developing arterial hypertension later in life.

The present study evaluated the association between maternal anthropometric measurements (weight, height and BMI) in pre-pregnancy and at the end of pregnancy (weight and height) and blood pressure levels among adolescent children at 11 years of age.

## Methods

Pelotas is a city in Southern Brazil, with approximately 340 thousand inhabitants [9]. In 1993, all children born in Pelotas and whose mothers resided in the urban area of the municipality at the time of delivery were eligible for participation in a longitudinal study of health. The main objective of the 1993 cohort was to evaluate time trends in health indicators throughout the lifespan, through a comparison with results of the 1982 study cohort [10] carried out in the same city. Of the 5,320 births, 16 mothers (0.3%) either could not be located or refused to participate in the study and 55 were stillbirths, resulting in a total enrollment of 5,249 live births. In 2004 and early 2005, we attempted to trace all cohort members, then in early adolescence, for a follow-up study [11]. We obtained written consent of adolescents and their mothers. The research project was approved by the Ethics Committee of the Federal University of Pelotas School of Medicine.

Interviewers (around 15 female graduate students aged ≥ 20 years old) were trained for two weeks in the application of a standardized and pre-tested questionnaire as also in the measurement of weight and height. Standardization sessions were carried out before the start of data collection and every month during the fieldwork. Approximately 10-15% of subjects were revisited, and a short version of the questionnaire was administered for quality control purposes. In addition, 35-50% of those not revisited at home and who had a home telephone answered this same short questionnaire by telephone [10,11].

The dependent variables investigated were systolic and diastolic blood pressure (DBP) of adolescent subjects, defined as the arithmetic mean of two measurements carried out at a 60-second interval using an OMRON HEM-629 portable digital wrist monitor (margin of error: 3 mmHg). This monitor has been validated previously and the authors' conclusion was: "the wrist monitor is not only easy to use, but also produces results very similar to those obtained by the standard indirect method" [12].

As independent variables, we obtained the following maternal anthropometric measures: a) prepregnancy weight - self-reported in the hospital interview (in case the mother was unable to provide this information, the first weight entry in the mother's pregnancy card was used); b) maternal height and weight at the end of pregnancy - determined at the time of admission by the team of interviewers, which included physicians, residents, and medical students [13]; c) prepregnancy body mass index (BMI) - estimated based on maternal prepregnancy weight and height.

Analysis was stratified by sex of the adolescent. For the analysis of the association between maternal weight at the end of pregnancy and the adolescent's blood pressure, we excluded all twin pregnancies (81 records). For comparison of mean SBP and DBP according to maternal anthropometric measures during gestation, we used one-way ANOVA test for homogeneous variances and ANOVA test for linear trend in crude analysis. In multivariate analysis, we used multiple linear regressions to adjust for potential confounders collected during the perinatal visit; these included family income at birth (Brazilian minimum wages in 1993 or ~US$150 per month), smoking and alcohol intake, both during pregnancy and gestational arterial hypertension [14]. We also included skin color, self-reported by the adolescent during the 2004-2005 follow-up [15]. We maintained in the analysis model all variables with significance level ≤ 0.20, as well as those regarded as confounders in the literature on the subject, even when these did not reach the indicated level of significance. To determine the magnitude of the effect of independent variables on adolescent blood pressure, the analysis was controlled, in a first model, for perinatal mediators (weight and length at birth) and in a second model for the adolescent's BMI, while maintaining the above-mentioned confounders in the analysis. All analyses were carried out using STATA 9.2 software.

## Results

Table 1 shows the distribution of adolescents traced at 11 years of age (n = 4,452) compared to the entire population of live births from 1993, according to maternal sociodemographic and anthropometric characteristics. There were no statistically significant differences for most variables studied, with the exception of family income at birth (≥ 6.1 category) and prepregnancy BMI (20.0 to 24.9 kg/m^2 ^category), for which the follow-up rate was lower.

**Table 1:** Description of the Original Population and of the Population Traced at age 11 Years.

Variable	Original population	Traced population*	**p-value**^**†**^
	n (%)	%	
**Sex**			0.17
Male	2606 (49.7)	86.9	
Female	2642 (50.3)	88.1	
**Family income (m.w.)**^**‡ **^**(1993)**			< 0.001
≥ 6.1	818 (15.6)	80.0	
3.1 - 6	1204 (22.9)	88.9	
1.1 - 3	2260 (43.1)	88.7	
≤ 1	967 (18.4)	88.3	
**Maternal smoking (pregnancy)**			0.3
No	3497 (66.6)	87.1	
Yes	1752 (33.5)	88.2	
**Maternal alcohol intake (pregnancy)**			0.8
No	4982 (94.9)	87.5	
Yes	267 (5.1)	86.9	
**Gestational arterial hypertension**			0.5
No	4337 (84.3)	87.7	
Yes	806 (15.7)	86.9	
**Maternal prepregnancy weight (kg)**			0.3
< 49	806 (15.7)	87.6	
49 - 53.9	1064 (20.7)	87.3	
54 - 60.9	1547 (30.1)	86.7	
≥ 61	1722 (33.5)	88.8	
**Maternal weight (end of pregnancy) (kg)**			0.7
< 55	339 (6.7)	87.9	
55 - 64.9	1426 (28.1)	88.1	
65 - 74.9	1776 (35.0)	86.9	
≥ 75	1527 (30.1)	88.3	
**Maternal height (end of pregnancy) (cm)**			0.09
≥ 165	1334 (25.4)	85.4	
160 - 164	1429 (27.2)	88.4	
155 - 159	1325(25.2)	87.9	
150 - 154	922 (17.6)	88.6	
< 150	239 (4.6)	84.5	
**Prepregnancy BMI**^**§ **^**(kg/m**^**2**^**)**			0.004
< 20.0	1147 (22.5)	87.6	
20.0 to 24.9	2811 (55.2)	86.6	
25.0 to 29.9	894 (17.5)	90.3	
30.0 or more	245 (4.8)	92.2	

1993 Cohort, 2004-05 Follow-up (Pelotas, Southern Brazil)

* Includes 141 deaths: 87.5% of cohort members traced at 11 years of age.

^† ^p-value from Chi-squared test for heterogeneity.

^‡ ^m.w - Brazilian minimum wages in 1993.

^§ ^BMI - indicates body mass index.

Mean (standard deviation; SD) SBP and DBP were 101.9 mm Hg (SD 12.3) and 63.4 mm Hg (DP 9.9), respectively, with no significant differences between sexes. Table 2 presents mean (SD) SBP and DBP stratified by sex. Among male adolescents, there was no significant association between means SBP or DBP and skin color or maternal alcohol intake during pregnancy. Among females, however, mean blood pressure was higher among subjects with black skin color, and lower among those whose mothers reported alcohol intake during pregnancy. Reported gestational arterial hypertension was directly associated with mean SBP among males, but was not associated with either SBP or DBP among females. Family income and maternal smoking during pregnancy were not associated with blood pressure in adolescents of either sex.

**Table 2:** Mean Blood Pressure (mm Hg) and Standard Deviation (SD) for the Total Sample, According to Independent Variables.

	Systolic blood pressure - Mean (SD)				Diastolic blood pressure - Mean (SD)			
	
	Males	p-value	Females	p-value	Males	p-value	Females	p-value
	(n = 2154)		(n = 2280)		(n = 2154)		(n = 2280)	
**Adolescent's skin color**		0.53*		< 0.004*		0.59*		< 0.001^†^
White	101.9 (12.1)		100.8 (12.3)		63.4 (9.8)		62.5 (9.5)	
Mulatto/Brown	102.5 (12.7)		102.7 (12.9)		64.0 (9.4)		64.5 (10.3)	
Black	102.7 (11.6)		103.3 (12.9)		64.1 (10.5)		4.6 (10.7)	
**Family income (m.w.)**^**§ **^**(1993)**		0.34^‡^		0.79^‡^		0.97^‡^		0.74^‡^
≥ 6.1	102.9 (11.6)		100.8 (12.5)		63.7 (10.0)		63.0 (9.6)	
3.1 - 6	102.4 (12.4)		102.4 (12.1)		63.6 (9.7)		63.5 (10.0)	
1.1 - 3	101.7 (12.3)		101.1 (12.4)		63.4 (9.9)		62.9 (9.7)	
≤ 1	102.5 (12.0)		101.9 (12.8)		63.9 (9.6)		63.2 (10.5)	
**Maternal smoking (pregnancy)**		0.79*		0.76*		0.64*		0.89*
No	102.2 (12.1)		101.6 (12.3)		63.6 (9.7)		63.1 (9.9)	
Yes	102.1 (12.2)		101.4 (12.7)		63.4 (10.0)		63.1 (9.8)	
**Maternal alcohol intake (pregnancy)**		0.75*		< 0.001*		0.39*		0.01*
No	102.2 (12.1)		101.8 (12.4)		63.5 (9.8)		63.3 (9.8)	
Yes	102.6 (12.8)		97.5 (13.2)		64.4 (9.3)		60.8 (10.6)	
**Gestational arterial hypertension**		0.005*		0.51*		0.15*		0.79*
No	101.9 (12.0)		101.4 (12.4)		63.5 (9.8)		63.1 (9.9)	
Yes	103.9 (12.9)		101.9 (13.1)		64.3 (10.3)		63.2 (9.9)	
**Maternal prepregnancy weight (kg)**		< 0.001^‡^		< 0.001^‡^		< 0.001^‡^		< 0.001^‡^
1^st ^quartile (lowest)	100.7 (12.0)		99.3 (12.0)		62.3 (9.5)		61.8 (9.7)	
2^nd ^quartile	101.9 (12.1)		101.1 (12.4)		63.5 (9.4)		62.5 (9.8)	
3^rd ^quartile	102.3 (12.3)		102.0 (12.4)		63.6 (10.1)		63.2 (10.0)	
4^th ^quartile (highest)	103.8 (12.0)		103.7 (12.7)		64.7 (9.9)		65.2 (9.8)	
**Maternal weight (end of pregnancy) (kg)**		< 0.001^‡^		< 0.001^‡^		< 0.001^‡^		< 0.001^‡^
1^st ^quartile (lowest)	100.8 (12.2)		100.1 (12.4)		62.4 (9.4)		61.9 (9.9)	
2^nd ^quartile	101.9 (11.9)		100.4 (11.9)		63.0 (9.6)		62.3 (9.5)	
3^rd ^quartile	102.3 (11.6)		102.6 (12.1)		64.2 (9.7)		63.6 (9.6)	
4^th ^quartile (highest)	103.7 (12.4)		103.4 (12.9)		64.5 (9.9)		64.8 (10.0)	
**Maternal height (end of pregnancy) (cm)**		0.02^‡^		0.02^‡^		0.58^‡^		0.005^‡^
1^st ^quartile (lowest)	101.4 (12.3)		100.8 (12.6)		63.4 (9.6)		62.3 (10.1)	
2^nd ^quartile	102.2 (12.4)		101.1 (11.9)		63.7 (9.9)		62.9 (9.5)	
3^rd ^quartile	102.6 (11.8)		102.3 (12.5)		63.7 (9.8)		63.6 (9.6)	
4^th ^quartile (highest)	103.1 (12.0)		102.3 (12.9)		63.7 (9.9)		63.9 (10.3)	
**Prepregnancy BMI (kg/m**^**2**^**)**		< 0.001^‡^		< 0.001^‡^		< 0.001^‡^		< 0.001^‡^
1^st ^quartile (lowest)	100.9 (11.5)		99.6 (11.8)		62.4 (9.3)		61.7 (9.2)	
2^nd ^quartile	101.7 (12.1)		100.8 (12.4)		63.2 (9.9)		62.6 (10.1)	
3^rd ^quartile	102.9 (12.8)		101.9 (12.2)		64.1 (9.7)		63.4 (9.7)	
4^th ^quartile (highest)	103.3 (12.1)		103.7 (12.9)		64.7 (10.0)		64.8 (10.2)	

1993 Cohort, 2005-05 Follow-up (Pelotas, Southern Brazil)

* p-value from One-way ANOVA for homogeneity variances.

^† ^p-value from Kruskall-Wallis test for heterogeneity variables.

^‡ ^p-value from ANOVA for linear trend.

^§ ^m.w. - Brazilian minimum wages in 1993.

With the exception of maternal height, which was not associated with DBP among males - all remaining maternal anthropometric variables showed statistically significant direct associations among subjects of both sexes. Associations also showed linear trends, which strengthens the hypothesis of a causal relationship given the biological gradient observed.

Additional files 1 and 2 Table S1 and Table S2 present the results of crude and adjusted linear regression analysis for SBP and DBP in relation to anthropometric variables for males and females, respectively.

Regression coefficients for the relationship between maternal prepregnancy weight and BMI, and weight at the end of pregnancy were positively associated with adolescent SBP and DBP among males, both in crude and adjusted analysis (Additional file 1 Table S1). The linear regression coefficient showed a difference in SBP of up to 3 mmHg between the male children of mothers in the highest and lowest quartiles of maternal prepregnancy weight, in crude analysis. For DBP, there was an increase in the linear regression coefficient of up to 2.4 mm Hg for this same comparison, also among males. Confounder control led to a slight reduction in regression coefficients, but statistical significance was maintained (Additional file 1 Table S1).

There was a positive association between SBP and maternal height among males. This association remained significant after confounder control in adjusted analysis. DBP, on the other hand, showed no association with maternal height in neither crude nor adjusted analyses (Additional File 1 Table S1).

The largest increase in linear regression coefficient was that seen for the highest quartile of maternal prepregnancy weight, for subjects of both sexes (Additional Files 1 and 2 Table S1 and Table S2).

Among females (Additional File 2 Table S2), there was a direct and significant association between SBP and DBP linear regression coefficients and all maternal anthropometric variables in both crude and adjusted analyses. It is worth noting that, the magnitude of increases in regression coefficients was greater among females than among males; increases of about 4.5 mm Hg in SBP between the highest and lowest maternal prepregnancy weight quartiles were observed in both crude and adjusted analyses. The corresponding increase in DBP was of almost 3.5 mm Hg. After confounder control, there was a smaller increase in linear regression coefficients for maternal prepregnancy weight and end of pregnancy for both SBP and DBP, with a reduction in coefficients for maternal height and prepregnancy BMI, while statistical significance was maintained (Additional File 2 Table S2).

In addition to confounder control, adjustment for mediators such as subject's weight and length at birth, as well as BMI at 11 years of age were also performed (Additional File 3 Table S3). Statistical significance was defined as a p-value below 0.05. In the first model, adjusted for weight and length at birth, associations between maternal anthropometric variables during pregnancy and SBP and DBP of male adolescents remained significant, with the exception of maternal height, which was also not associated with the DBP in the previous analysis. Adjustment for current adolescent's BMI led to loss of significance of all associations among male adolescents. A similar pattern was seen among females, namely, in the first model the linear regression coefficients for SBP and DBP remained associated with maternal weight before and at the end of pregnancy as well as with prepregnancy BMI. There was also a significant direct association among females between DBP and maternal height.

Adjustment for current adolescent's BMI revealed certain differences between males and females. Whereas associations between SBP and maternal anthropometric variables were lost, the association between DBP and maternal prepregnancy weight maintained a borderline association (*p *= 0.05).

## Discussion

Maternal anthropometric variables - prepregnancy weight and BMI and weight at the end of pregnancy - showed independent positive associations with SBP and DBP among 11-year-olds of both sexes. This is the first study which investigates this association in a developing country.

The fact that this is a population-based cohort with a high follow-up rate (87.5%) allows us to extrapolate our present results to the population of the city of Pelotas. Another positive feature of this study is the standardization of measurements of maternal anthropometric variables at the end of pregnancy and of adolescent weight and height.

One of the limitations of the present study is the validity of pre-gestational information, since this data was either self-reported by the mother or obtained from the mother's pregnancy card. Stevens-Simon et al [16] and de Oliveira et al [17] found high Pearson correlation coefficients between measured and self-reported maternal prepregnancy weight (r = 0.96 for both). However, a study by Yu et al [18] detected a statistically significant difference of up to 3 kg less for self-reported when compared to estimated weight. Therefore, the possibility of information bias in analyses of self-reported maternal weight cannot be discarded.

Another methodological aspect to be discussed is the use of digital wrist monitors for measuring blood arterial pressure. Although the gold-standard for this measurement is the mercury sphygmomanometer, previously published evaluations of digital monitors emphasize their ease of use in population-based settings and the similarity between results obtained using this equipment and the gold-standard [12,19].

The finding of higher SBP among children of mothers who were heavier at the beginning of pregnancy is in agreement with current literature. Jarvelin et al [20] evaluated 5,960 subjects aged 31 years in Finland and found that, irrespective of sex, mean SBP was higher among subjects whose mothers were in the highest tercile of prepregnancy weight. Though a similar trend was observed for DBP, this association did not reach statistical significance [20]. Lawor et al [21], in a study of 3,864 5-year-olds from Australia, found that for each standard-deviation increase of maternal prepregnancy weight - equivalent to approximately 11.4 kg - there was a 0.77 mm Hg increase in the child's SBP. Similar findings were reported by Laor et al [4] in Israel (10,883 17-year-old subjects) and Bergel et al [22] in Argentina (518 children aged 5-9 years); in both studies, the associations lost statistical significance in adjusted analysis, probably due to the inclusion of prepregnancy BMI into the model.

Regarding maternal weight at the end of pregnancy, adolescents of both sexes whose mothers were heavier at the end of pregnancy showed higher SBP than the children of leaner mothers. We found no other reports of this association in any literature.

Another important finding in the present study is that both SBP and DBP of adolescents whose mothers had higher prepregnancy BMI were higher than among those whose mothers had lower prepregnancy BMI. Laor et al [4] found a similar association; however, statistical significance was lost after adjustment for maternal and perinatal confounders. Lawlor et al [21], in an analysis of the effect of child age and sex, found that for each standard deviation increase in maternal prepregnancy BMI - roughly equivalent to 4 kg/m^2 ^- SBP increased by 0.78 mm Hg. Although this difference decreased to 0.38 mm Hg after adjustment for maternal (perinatal) and child characteristics, the association between maternal prepregnancy BMI and SBP in children remained significant.

Adolescent children of taller mothers had higher SBP levels than the children of shorter mothers. Although a similar association was detected for DBP, this association was restricted to female adolescents. A longitudinal study carried out in the city of Avon, England, (1,860 3-year-old subjects) showed that for every 10 cm increase in maternal height, SBP and DBP increased 1.2 and 0.6 mm Hg, respectively (*p *= 0.003); however, after adjustment for the child's height, the strength of the association dropped markedly to 0.3 and 0.4 mm Hg, respectively (*p *= 0.05) [23].

Lawor et al [21] found no association between maternal height and SBP in crude analysis; however, after allowing for the effects of subject sex and age and maternal characteristics, the authors detected an increase in SBP of 0.41 mmHg for each standard deviation increase in maternal height (6.5 cm); subsequently, after adjustment for birthweight, weight and height at 5 years of age, the authors reported a reduction in SBP [18]. In the Cebu Longitudinal Health and Nutrition Survey (CLHNS), which followed 2,026 adolescents aged 14-16 years, the authors report that for each one-centimeter increase in maternal height, SBP decreased by 0.16 mmHg among male adolescents, after adjustment for weight and length at birth, adolescent's anthropometric characteristics, and maternal diet, blood pressure and anthropometric variables during pregnancy [5]. Barker et al [1], studying a population of 3,259 adults, found that SBP among children of taller mothers was 2.7 mm Hg lower than the ones of shorter mothers, after adjustment for subject's weight.

Interpreting findings from the literature on the subject of maternal anthropometry and blood pressure of adolescents requires caution, given the fact that several studies fail to account for certain potential confounders and mediators. In the present study, confounders such as adolescent's skin color, family income, maternal smoking, reported alcohol intake and gestational arterial hypertension during pregnancy were accounted for when testing the association between maternal anthropometry and adolescent blood pressure, leading to no loss of statistical significance of our findings. It should be noted that adjustment for mediators such as maternal and perinatal characteristics led to a reduction in the magnitude of regression coefficients, and that including the adolescent's BMI in the model either led to a loss of statistical significance or to a borderline association. Variation in blood pressure during adolescence can be explained in part by maternal anthropometric variables; however the adolescent's BMI is an important determinant, since it reflects both genetic predisposition to obesity and environmental influences after birth throughout life [8,24].

The hypothesis of the fetal origin of diseases suggests that intrauterine aggression can lead to abnormal adaptations in the various systems of the fetus [25]. Despite the evidence that maternal undernutrition during pregnancy may constitute one such aggression, it is possible that obesity may also be included in this category. It has been shown that obese women can gain very little weight - or even lose weight - during pregnancy, which may lead to a deficit in fetal growth [26]. Regarding both extremes of malnutrition as risk factors for high blood pressure in adolescence, it is conceivable that increases in blood pressure are secondary to deficiencies in macro or micronutrients in the pregnant mother's diet [27].

An alternative hypothesis is that both maternal overweight and blood pressure in adolescence are associated to socio-economic status (SES). The strong association between skin color and blood pressure (Table 2) is very suggestive. Family income is not consistently associated with BP; but this might be a result of misclassification or that there is not enough variability in this marker in the population under study. We did not incorporate any additional SES variable other than family income in the analysis.

## Conclusions

In conclusion, our findings indicate the presence of a direct association between maternal prepregnancy weight and BMI and weight at the end of pregnancy with high SBP among adolescents, irrespective of sex. We also found a direct association between maternal height and SBP in adolescents of both sexes, and with DBP among females only.

We underscore the importance of evaluating the nutritional status of women of reproductive age - with emphasis primarily on the population of pregnant women with tendency towards overweight - in order to program early interventions targeting diet and physical activity level. Likewise, we suggest periodic monitoring of nutritional status and blood pressure during childhood and adolescence, given that detection of high blood pressure levels can allow for timely intervention aiming to reduce the potential effects of arterial hypertension later in life.

## Competing interests

The authors declare that they have no competing interests.

## Authors' contributions

AMM supervised the study and collaborated in writing the manuscript. HC and RBN contributed to data analysis and to writing the manuscript. PH and CA participated in the revision of the manuscript. All authors read and approved the final manuscript.

## Pre-publication history

The pre-publication history for this paper can be accessed here:

http://www.biomedcentral.com/1471-2458/10/434/prepub

## Supplementary Material

Additional file 1 **Crude and Adjusted Linear Regression for Systolic and Diastolic Blood Pressure Among Males, According to Maternal Anthropometric Variables**. 1993 Cohort, 2005-05 Follow-up (Pelotas, Southern Brazil). Table S1 Click here for file

Additional file 2 **Crude and Adjusted Linear Regression for Systolic and Diastolic Arterial Pressure Among Females, According to Maternal Anthropometric Variables**. 1993 Cohort, 2005-05 Follow-up (Pelotas, Southern Brazil). Table S2. Click here for file

Additional file 3 **Linear regression for Systolic and Diastolic Arterial Pressure, Crude and Adjusted for Mediating Factors**. 1993 Cohort, 2005-05 Follow-up (Pelotas, Southern Brazil). Table S3. Click here for file
