# Supplementary material for: Maternal anthropometric characteristics in pregnancy and blood pressure among adolescents: 1993 live birth cohort, Pelotas, southern Brazil
Source: BMC Public Health. 2010 Jul 23;10:434. doi: 10.1186/1471-2458-10-434 (PMC2918557; doi:10.1186/1471-2458-10-434)
Supplement: Additional file 1 — Crude and Adjusted Linear Regression for Systolic and Diastolic Blood Pressure Among Males, According to Maternal Anthropometric Variables. 1993 Cohort, 2005-05 Follow-up (Pelotas, Southern Brazil). Table S1 [file 1471-2458-10-434-S1.DOC]

| **Table 3.** Crude and Adjusted Linear Regression for Systolic and Diastolic Blood Pressure Among Males, According to Maternal Anthropometric Variables. 1993 Cohort, 2005-05 Follow-up (Pelotas, Southern Brazil) | | | | | | | | |
| --- | --- | --- | --- | --- | --- | --- | --- | --- |
| **Variables** | **Linear regression coefficients (standard error)** | | | | | | | |
|  | **Systolic blood pressure (mm Hg)**  **(n = 2154 )** | | | | **Diastolic blood pressure (mm Hg)**  **(n = 2154 )** | | | |
|  | **Crude** | **p-value^*^** | **Adjusted^†^** | **p-value^*^** | **Crude** | **p-value^*^** | **Adjusted^†^** | **p-value^*^** |
| **Maternal prepregnancy weight (kg)** |  | <0.001 |  | <0.001 |  | <0.001 |  | <0.001 |
| 1^st^ quartile (lowest) | 0.00 |  | 0.00 |  | 0.00 |  | 0.00 |  |
| 2^nd^ quartile | 1.29 (0.72) |  | 1.11 (0.73) |  | 1.21 (0.58) |  | 1.19 (0.59) |  |
| 3^rd^ quartile | 1.60 (0.77) |  | 1.48 (0.79) |  | 1.31 (0.62) |  | 1.33 (0.64) |  |
| 4^th^ quartile (highest) | 3.09 (0.72) |  | 2.82 (0.76) |  | 2.40 (0.58) |  | 2.31 (0.61) |  |
| **Maternal weight (end of pregnancy) (kg)** |  | <0.001 |  | 0.001 |  | <0.001 |  | <0.001 |
| 1^st^ quartile (lowest) | 0.00 |  | 0.00 |  | 0.00 |  | 0.00 |  |
| 2^nd^ quartile | 1.06 (0.76) |  | 0.93 (0.78) |  | 0.59 (0.61) |  | 0.77 (0.63) |  |
| 3^rd^ quartile | 1.43 (0.73) |  | 1.26 (0.76) |  | 1.78 (0.58) |  | 1.84 (0.61) |  |
| 4^th^ quartile (highest) | 2.91 (0.73) |  | 2.62 (0.77) |  | 2.05 (0.59) |  | 2.08 (0.62) |  |
| **Maternal height (end of pregnancy) (cm)** |  | 0.02 |  | 0.02 |  | 0.58 |  | 0.49 |
| 1^st^ quartile (lowest) | 0.00 |  | 0.00 |  | 0.00 |  | 0.00 |  |
| 2^nd^ quartile | 0.72 (0.69) |  | 0.80 (0.71) |  | 0.25 (0.56) |  | 0.33 (0.58) |  |
| 3^rd^ quartile | 1.19 (0.73) |  | 1.30 (0.75) |  | 0.30 (0.59) |  | 0.36 (0.61) |  |
| 4^th^ quartile (highest) | 1.64 (0.78) |  | 1.76 (0.80) |  | 0.33 (0.63) |  | 0.45 (0.65) |  |
| **Prepregnancy BMI (kg/m^2^)** |  | <0.001 |  | 0.003 |  | <0.001 |  | <0.001 |
| 1^st^ quartile (lowest) | 0.00 |  | 0.00 |  | 0.00 |  | 0.00 |  |
| 2^nd^ quartile | 0.73 (0.75) |  | 0.51 (0.76) |  | 0.75 (0.60) |  | 0.67 (0.62) |  |
| 3^rd^ quartile | 1.94 (0.74) |  | 1.69 (0.76) |  | 1.68 (0.59) |  | 1.54 (0.61) |  |
| 4^th^ quartile (highest) | 2.35 (0.73) |  | 1.97 (0.76) |  | 2.30 (0.59) |  | 2.11 (0.61) |  |

* p-value from test for trend.

^†^ Adjusted for adolescent’s skin color, family income, and maternal smoking, alcohol ingestion, and gestational arterial hypertension during pregnancy
