# Supplementary material for: Maternal anthropometric characteristics in pregnancy and blood pressure among adolescents: 1993 live birth cohort, Pelotas, southern Brazil
Source: BMC Public Health. 2010 Jul 23;10:434. doi: 10.1186/1471-2458-10-434 (PMC2918557; doi:10.1186/1471-2458-10-434)
Supplement: Additional file 2 — Crude and Adjusted Linear Regression for Systolic and Diastolic Arterial Pressure Among Females, According to Maternal Anthropometric Variables. 1993 Cohort, 2005-05 Follow-up (Pelotas, Southern Brazil). Table S2. [file 1471-2458-10-434-S2.DOC]

| **Table 4.** Crude and Adjusted Linear Regression for Systolic and Diastolic Arterial Pressure Among Females, According to Maternal Anthropometric Variables. 1993 Cohort, 2005-05 Follow-up (Pelotas, Southern Brazil) | | | | | | | | |
| --- | --- | --- | --- | --- | --- | --- | --- | --- |
| **Variables** | **Linear regression coefficients (standard error)** | | | | | | | |
|  | **Systolic blood pressure (mm Hg)**  **(n = 2280)** | | | | **Diastolic blood pressure (mm Hg)**  **(n = 2280)** | | | |
|  | **Crude** | **p-value^*^** | **Adjusted^†^** | **p-value^*^** | **Crude** | **p-value^*^** | **Adjusted^†^** | **p-value^*^** |
| **Maternal prepregnancy weight (kg)** |  | <0.001 |  | <0.001 |  | <0.001 |  | <0.001 |
| 1^st^ quartile (lowest) | 0.00 |  | 0.00 |  | 0.00 |  | 0.00 |  |
| 2^nd^ quartile | 1.82 (0.73) |  | 1.90 (0.74) |  | 0.72 (0.58) |  | 0.79 (0.58) |  |
| 3^rd^ quartile | 2.67 (0.76) |  | 2.57 (0.78) |  | 1.43 (0.60) |  | 1.38 (0.61) |  |
| 4^th^ quartile (highest) | 4.39 (0.74) |  | 4.42 (0.76) |  | 3.39 (0.59) |  | 3.35 (0.60) |  |
| **Maternal weight (end of pregnancy) (kg)** |  | <0.001 |  | <0.001 |  | <0.001 |  | <0.001 |
| 1^st^ quartile (lowest) | 0.00 |  | 0.00 |  | 0.00 |  | 0.00 |  |
| 2^nd^ quartile | 0.36 (0.74) |  | 0.49 (0.75) |  | 0.37 (0.58) |  | 0.52 (0.59) |  |
| 3^rd^ quartile | 2.54 (0.75) |  | 2.60 (0.77) |  | 1.59 (0.59) |  | 1.69 (0.61) |  |
| 4^th^ quartile (highest) | 3.30 (0.74) |  | 3.38 (0.76) |  | 2.79 (0.59) |  | 2.81 (0.60) |  |
| **Maternal height (end of pregnancy) (cm)** |  | 0.02 |  | 0.04 |  | 0.005 |  | 0.02 |
| 1^st^ quartile (lowest) | 0.00 |  | 0.00 |  | 0.00 |  | 0.00 |  |
| 2^nd^ quartile | 0.27 (0.70) |  | 0.15 (0.71) |  | 0.59 (0.56) |  | 0.55 (0.56) |  |
| 3^rd^ quartile | 1.46 (0.72) |  | 1.30 (0.74) |  | 1.29 (0.57) |  | 1.21 (0.58) |  |
| 4^th^ quartile (highest) | 1.49 (0.79) |  | 1.31 (0.81) |  | 1.52 (0.62) |  | 1.34 (0.64) |  |
| **Prepregnancy BMI (kg/m^2^)** |  | <0.001 |  | <0.001 |  | <0.001 |  | <0.001 |
| 1^st^ quartile (lowest) | 0.00 |  | 0.00 |  | 0.00 |  | 0.00 |  |
| 2^nd^ quartile | 1.18 (0.75) |  | 0.88 (0.76) |  | 0.85 (0.60) |  | 0.62 (0.60) |  |
| 3^rd^ quartile | 2.34 (0.75) |  | 2.19 (0.76) |  | 1.67 (0.60) |  | 1.56 (0.60) |  |
| 4^th^ quartile (highest) | 4.07 (0.75) |  | 4.03 (0.76) |  | 3.03 (0.60) |  | 2.93 (0.60) |  |

* p-value from test for trend.

^†^ Adjusted for adolescent’s skin color, family income, and maternal smoking, alcohol intake, and gestational arterial hypertension during pregnancy.
