# Supplementary material for: Maternal anthropometric characteristics in pregnancy and blood pressure among adolescents: 1993 live birth cohort, Pelotas, southern Brazil
Source: BMC Public Health. 2010 Jul 23;10:434. doi: 10.1186/1471-2458-10-434 (PMC2918557; doi:10.1186/1471-2458-10-434)
Supplement: Additional file 3 — Linear regression for Systolic and Diastolic Arterial Pressure, Crude and Adjusted for Mediating Factors. 1993 Cohort, 2005-05 Follow-up (Pelotas, Southern Brazil). Table S3. [file 1471-2458-10-434-S3.DOC]

| **Table 5.** Linear regression for Systolic and Diastolic Arterial Pressure, Crude and Adjusted for Mediating Factors. 1993 Cohort, 2005-05 Follow-up (Pelotas, Southern Brazil) | | | | | | |
| --- | --- | --- | --- | --- | --- | --- |
| **Variables** | **Linear regression coefficients (standard error)** | | | | | |
|  | **Systolic blood pressure (mm Hg)** | | | **Diastolic blood pressure (mm Hg)** | | |
|  | **Crude** | **Adjusted^*^** | **Adjusted^†^** | **Crude** | **Adjusted^*^** | **Adjusted^†^** |
| **Males (n = 2154)** |  |  |  |  |  |  |
| **Maternal prepregnancy weight (kg)** | <0.001^‡^ | 0.001^‡^ | 0.83^‡^ | <0.001^‡^ | 0.001^‡^ | 0.37^‡^ |
| 1^st^ quartile (lowest) | 0.00 | 0.00 | 0.00 | 0.00 | 0.00 | 0.00 |
| 2^nd^ quartile | 1.29 (0.72) | 1.08 (0.74) | 0.49 (0.69) | 1.21 (0.58) | 1.19 (0.59) | 0.85 (0.58) |
| 3^rd^ quartile | 1.60 (0.77) | 1.47 (0.80) | 0.14 (0.76) | 1.31 (0.62) | 1.36 (0.65) | 0.60 (0.63) |
| 4^th^ quartile (highest) | 3.09 (0.72) | 2.64 (0.79) | -0.06 (0.76) | 2.40 (0.58) | 2.21 (0.64) | 0.68 (0.63) |
| **Maternal weight (end of pregnancy) (kg)** | <0.001^‡^ | 0.002^‡^ | 0.67^‡^ | <0.001^‡^ | 0.001^‡^ | 0.31^‡^ |
| 1^st^ quartile (lowest) | 0.00 | 0.00 | 0.00 | 0.00 | 0.00 | 0.00 |
| 2^nd^ quartile | 1.06 (0.76) | 0.95 (0.79) | -0.09 (0.74) | 0.59 (0.61) | 0.83 (0.64) | 0.23 (0.62) |
| 3^rd^ quartile | 1.43 (0.73) | 1.32 (0.78) | -0.16 (0.74) | 1.78 (0.58) | 1.90 (0.63) | 1.06 (0.61) |
| 4^th^ quartile (highest) | 2.91 (0.73) | 2.59 (0.83) | -0.34 (0.79) | 2.05 (0.59) | 2.09 (0.67) | 0.42 (0.66) |
| **Maternal height (end of pregnancy) (cm)** | 0.02^‡^ | 0.04^‡^ | 0.11^‡^ | 0.58^‡^ | 0.73^‡^ | 0.99^‡^ |
| 1^st^ quartile (lowest) | 0.00 | 0.00 | 0.00 | 0.00 | 0.00 | 0.00 |
| 2^nd^ quartile | 0.72 (0.69) | 0.76 (0.71) | 0.39 (0.67) | 0.25 (0.56) | 0.30 (0.58) | 0.09 (0.56) |
| 3^rd^ quartile | 1.19 (0.73) | 1.10 (0.76) | 0.86 (0.71) | 0.30 (0.59) | 0.14 (0.62) | -0.0001 (0.59) |
| 4^th^ quartile (highest) | 1.64 (0.78) | 1.65 (0.81) | 1.09 (0.76) | 0.33 (0.63) | 0.34 (0.66) | 0.01 (0.64) |
| **Prepregnancy BMI (kg/m^2^)** | <0.001^‡^ | 0.01^‡^ | 0.26^‡^ | <0.001^‡^ | 0.001^‡^ | 0.37^‡^ |
| 1^st^ quartile (lowest) | 0.00 | 0.00 | 0.00 | 0.00 | 0.00 | 0.00 |
| 2^nd^ quartile | 0.73 (0.75) | 0.46 (0.77) | -0.29 (0.72) | 0.75 (0.60) | 0.66 (0.62) | 0.23 (0.60) |
| 3^rd^ quartile | 1.94 (0.74) | 1.64 (0.77) | 0.18 (0.72) | 1.68 (0.59) | 1.58 (0.62) | 0.76 (0.60) |
| 4^th^ quartile (highest) | 2.35 (0.73) | 1.75 (0.78) | -0.07 (0.75) | 2.30 (0.59) | 1.99 (0.63) | 0.40 (0.63) |
| **Females (n = 2280)** |  |  |  |  |  |  |
| **Maternal prepregnancy weight (kg)** | <0.001^‡^ | <0.001^‡^ | 0.21^‡^ | <0.001^‡^ | <0.001^‡^ | 0.05^‡^ |
| 1^st^ quartile (lowest) | 0.00 | 0.00 | 0.00 | 0.00 | 0.00 | 0.00 |
| 2^nd^ quartile | 1.82 (0.73) | 1.79 (0.75) | 0.47 (0.70) | 0.72 (0.58) | 0.73 (0.59) | -0.13 (0.57) |
| 3^rd^ quartile | 2.67 (0.76) | 2.43 (0.79) | 0.46 (0.74) | 1.43 (0.60) | 1.30 (0.63) | 0.07 (0.60) |
| 4^th^ quartile (highest) | 4.39 (0.74) | 4.21 (0.78) | 0.98 (0.75) | 3.39 (0.59) | 3.23 (0.62) | 1.14 (0.61) |
| **Maternal weight (end of pregnancy) (kg)** | <0.001^‡^ | <0.001^‡^ | 0.92^‡^ | <0.001^‡^ | <0.001^‡^ | 0.25^‡^ |
| 1^st^ quartile (lowest) | 0.00 | 0.00 | 0.00 | 0.00 | 0.00 | 0.00 |
| 2^nd^ quartile | 0.36 (0.74) | 0.19 (0.77) | -1.01 (0.71) | 0.37 (0.58) | 0.40 (0.61) | -0.36 (0.58) |
| 3^rd^ quartile | 2.54 (0.75) | 2.38 (0.79) | 0.20 (0.74) | 1.59 (0.59) | 1.59 (0.63) | 0.24 (0.60) |
| 4^th^ quartile (highest) | 3.30 (0.74) | 3.12 (0.81) | -0.35 (0.77) | 2.79 (0.59) | 2.73 (0.64) | 0.55 (0.63) |
| **Maternal height (end of pregnancy) (cm)** | 0.02^‡^ | 0.11^‡^ | 0.37^‡^ | 0.005^‡^ | 0.03^‡^ | 0.09^‡^ |
| 1^st^ quartile (lowest) | 0.00 | 0.00 | 0.00 | 0.00 | 0.00 | 0.00 |
| 2^nd^ quartile | 0.27 (0.70) | 0.07 (0.71) | -0.32 (0.65) | 0.59 (0.56) | 0.49 (0.56) | 0.24 (0.53) |
| 3^rd^ quartile | 1.46 (0.72) | 0.99 (0.74) | 0.79 (0.68) | 1.29 (0.57) | 1.08 (0.59) | 0.97 (0.56) |
| 4^th^ quartile (highest) | 1.49 (0.79) | 1.02 (0.82) | 0.26 (0.75) | 1.52 (0.62) | 1.23 (0.65) | 0.76 (0.62) |
| **Prepregnancy BMI (kg/m^2^)** | <0.001^‡^ | <0.001^‡^ | 0.17^‡^ | <0.001^‡^ | <0.001^‡^ | 0.10^‡^ |
| 1^st^ quartile (lowest) | 0.00 | 0.00 | 0.00 | 0.00 | 0.00 | 0.00 |
| 2^nd^ quartile | 1.18 (0.75) | 0.71 (0.77) | -0.20 (0.71) | 0.85 (0.60) | 0.51 (0.61) | -0.06 (0.58) |
| 3^rd^ quartile | 2.34 (0.75) | 2.11 (0.77) | 0.05 (0.72) | 1.67 (0.60) | 1.50 (0.61) | 0.19 (0.59) |
| 4^th^ quartile (highest) | 4.07 (0.75) | 3.85 (0.78) | 0.98 (0.74) | 3.03 (0.60) | 2.80 (0.62) | 0.94 (0.60) |

* Adjusted for weight and length at birth.

† Adjusted for weight and length at birth and adolescent’s body mass index.

‡ p-value from test for trend
